# Morphology of the first instar larva of obligatory traumatic myiasis agents (Diptera: Calliphoridae, Sarcophagidae)

**DOI:** 10.1007/s00436-014-3808-x

**Published:** 2014-02-20

**Authors:** K. Szpila, M. J. R. Hall, A. H. Wardhana, T. Pape

**Affiliations:** 1 Chair of Ecology and Biogeography, Faculty of Biology and Environmental Protection, Nicolaus Copernicus University, Lwowska 1, Toruń, 87-100 Poland; 2 Department of Life Sciences, Natural History Museum, Cromwell Road, London, SW7 5BD UK; 3 Department of Parasitology, Indonesian Research Centre for Veterinary Science, JL. Martadinata 30, Bogor, West Java Indonesia; 4 Natural History Museum of Denmark, University of Copenhagen, Universitetsparken 15, Copenhagen, 2100 Denmark

**Keywords:** Obligatory traumatic myiasis agents, *Chrysomya bezziana*, *Cochliomyia hominivorax*, *Wohlfahrtia magnifica*, First instar larva, Morphology, Identification, SEM

## Abstract

There are only three fly species that are obligate agents of traumatic myiasis of humans and livestock: a single species of flesh fly, *Wohlfahrtia magnifica* (Sarcophagidae), and two species of blow flies, *Chrysomya bezziana* and *Cochliomyia hominivorax* (Calliphoridae). The morphology of their first instar larvae is thoroughly and consistently documented here with light microscopy photographs and scanning electron microscopy micrographs. The following morphological structures are documented: pseudocephalon, antennal complex, maxillary palpus, oral ridges, thoracic and abdominal spinulation, spiracular field, posterior spiracles and cephaloskeleton. New diagnostic features drawn from the cephaloskeleton and the spinulation of abdominal segments, including the anal pad, are discovered and extensively described. Earlier descriptions in the literature are revisited, and major discrepancies between these and the results of the current study are discussed. The present results allow clarification, correction and, especially, complementation of information provided by earlier authors. The relatively distant taxonomic position of all three species is evidence that obligatory myiasis has arisen independently, and the extensively similar morphology in the first instar larvae of *Chrysomya bezziana*, *Cochliomyia hominivorax* and *W. magnifica* in comparison to necrophagous species, especially the enhancement of the anterior part of the cephaloskeleton and the segmental spinulation, is therefore best interpreted as homoplasic adaptations to a life strategy as obligate vertebrate parasites. An identification key for first instar larvae of all obligatory traumatic myiasis agents of mammals is provided.

## Introduction

Traumatic myiasis of domestic animals and humans is a serious parasitic condition causing high economic losses around the world in addition to the suffering of those afflicted (Hall and Wall 1995). This kind of myiasis can involve several species of blowflies and fleshflies, but among these, only three are considered to be obligatory traumatic myiasis agents (abbreviated below as OTMA), and care needs to be taken in their identification (Hall 2008). These three species, *Chrysomya bezziana* Villeneuve, 1914, *Cochliomyia hominivorax* (Coquerel, 1858) and *Wohlfahrtia magnifica* (Schiner, 1862) are obligatory parasites, which under natural conditions complete their larval development only in the tissues of living vertebrates (Spradbery 1994; Sotiraki et al. 2010).

Until the second half of the twentieth century, the geographical distribution of these three OTMAs did not overlap, with the presence of *Chrysomya bezziana* confined to the Oriental Region and sub-Saharan Africa, *Cochliomyia hominivorax* in the warmer parts of the New World and *W. magnifica* in southern areas of the Palaearctic Region (Spradbery 1994; Hall and Farkas 2000). This situation was changed by the human-assisted introduction of *Cochliomyia hominivorax* to North Africa and the expansion of *Chrysomya bezziana* into the southern parts of the Palaearctic Region (Spradbery 1994; Lindquist et al. 1992). *Cochliomyia hominivorax* was successfully eradicated from the Old World (Lindquist et al. 1992), but *Chrysomya bezziana* seems to be well established in at least some Middle East countries, e.g. Iran and Oman (Hall et al. 2009). The entire Mediterranean Region is an area of potential sympatry of all OTMA species (Sutherst et al. 1989).

According to their considerable economic and medical importance, OTMA species have been the subject of intense research into many aspects of their biology and morphology (e.g. Laake et al. 1936; Kitching 1976; Ruiz-Martinez et al. 1989, 1990). However, most contributions to larval morphology have been concentrated on third instar larvae, L3 (e.g. Knipling 1939; Zumpt 1965; Erzinçlioğlu 1987; Spradbery 2002; Hall 2008; Florez and Wolff 2009). Morphology of the first instar larva, L1, was omitted or treated superficially because it has a relatively short developmental period and, therefore, is rarely encountered during the collection of specimens from infested wounds (Hall 2008).

Descriptions and illustrations of the L1 of *Chrysomya bezziana* are available in the publications of Zumpt (1965), Kitching (1976), Gan (1980) and Spradbery (2002). None of these studies contains an elaborate documentation of larval morphology. Zumpt (1965) provided only a brief description of the larval habitus, and both Kitching (1976) and Spradbery (2002) gave somewhat superficial descriptions supported by a few scanning electron micrographs. Gan (1980) presented an excellent drawing of the cephaloskeleton accompanied by a very short description of this structure. The morphology of the L1 of *Cochliomyia hominivorax* has been better documented than that of *Chrysomya bezziana*, mostly thanks to Laake et al. (1936). This landmark paper was taken further by the contribution of Leite and Guevara (1993), where several SEM micrographs are used for illustrating a number of morphological characters, although the associated description is rather superficial. An important contribution was made by Lopes (1983), who presented a figure of the anterior part of the cephaloskeleton of the L1 of *Cochliomyia hominivorax*, with the addition of a few comments concerning cephaloskeleton diversity in Calliphoridae and Sarcophagidae. Details of the morphology of the L1 of *W. magnifica* were presented by Valentyuk (1971) and Schumann et al. (1976). Lehrer and Fromunda (1986) provided a detailed and fully illustrated description, but this was largely restricted to the cephaloskeleton. Line drawings, detailed descriptions and SEM micrographs of larvae of *W. magnifica* were included in two papers of Ruiz-Martinez et al. (1989, 1990). However, for the L1, the SEM documentation was restricted to the exposed parts of the mouthhooks and labrum, as well as the spines of the anterior thoracic segments (Ruiz-Martinez et al. 1990), and their interpretations and terminology are partly conflicting with what is presented here.

Recent studies of larval morphology of higher Diptera have introduced high standards of documentation (e.g. Grzywacz et al. 2012; Klong-klaew et al. 2012; Sanit et al. 2012; Semelbauer and Kozánek 2012; Szpila et al. 2013; Velasquez et al. 2013), and it is appropriate and timely to revise the available morphological documentation of such medically and economically important species as the OTMAs to a similar high quality. Until now, no identification key to the L1 of all three OTMA species has been published.

The objectives of the present paper were (1) to revise existing data concerning the L1 morphology of the known OTMAs, (2) to expand this knowledge by providing new data and (3) to prepare an easily applied identification key to the L1 of all OTMAs.

## Material and methods

Larvae of *Chrysomya bezziana* and *Cochliomyia hominivorax* were obtained from laboratory colonies maintained at the Indonesian Research Centre for Veterinary Science, Bogor, Indonesia, and the Mexican-American Commission for the Eradication of Screwworm (COMEXA), Chiapa de Corzo, Mexico, respectively. Larvae of *W. magnifica* were obtained from gravid females collected from rural surveys around Budapest, Hungary (Hall et al. 2009).

For all species, live larvae were killed by immersion for 30 s in hot water (about 80–95 °C) to extend the pseudocephalon and avoid subsequent deformation when stored in 70 % ethanol (Adams and Hall 2003).

Ten L1 of each species were slide-mounted in Hoyer’s medium (Cielecka et al. 2009) for light microscopy. A Nikon 8400 digital camera mounted on a Nikon Eclipse E200 microscope (Nikon Corporation, Tokyo, Japan) was used for photomicrography. Another ten L1 of each species were prepared for SEM by dehydration through 80, 90 and 99.5 % ethanol, critical point dried in CO_2_ and sputter-coated with platinum. SEM images were taken with a JEOL JSM-6335F scanning electron microscope (JEOL Ltd., Tokyo, Japan).

Morphological terminology follows Courtney et al. (2000) and Szpila and Villet (2011). In particular, for the sensory cluster in the central part of the maxillary palpus, Szpila et al. (2008) classified the most prominent six sensilla as three sensilla coeloconica and three sensilla basiconica. This may be too generalised as sb3 deviates by being a composite structure of a small sensilla associated with an ear-like fold of the integument. We maintain the terminology of Szpila et al. (2008) acknowledging that more elaborate studies (e.g. histological, developmental genetic) may bring evidence that leads to an improved terminology.

## Results

### Redescriptions of the first instar larva of *Chrysomya bezziana*, *Cochliomyia hominivorax* and *Wohlfahrtia magnifica*

The L1 of all OTMAs possess the general habitus characteristic for most Calyptratae, being divided into a bilobed pseudocephalon (termed pc), three thoracic segments (t1–t3), seven abdominal segments (a1–a7) and an anal division (ad), which carries the posterior spiracles (Fig. 7a–c). Each thoracic segment has a pair of Keilin’s organs located ventrally (Figs. 2g and 4f). Laterally on t1, close to the posterior edge of the segment, is located the simple aperture of the anterior spiracle, usually overlooked in descriptions of L1 (Fig. 4a). Each of a1–a7 and ad has a transverse crevice on the ventral surface and lateral creeping welts (Fig. 3a). Specific morphological details are described separately for each species.

#### *Chrysomya bezziana* Villeneuve, 1914 (Figs. 1a–g; 6a, b; and 7a)

**Fig. 1:**
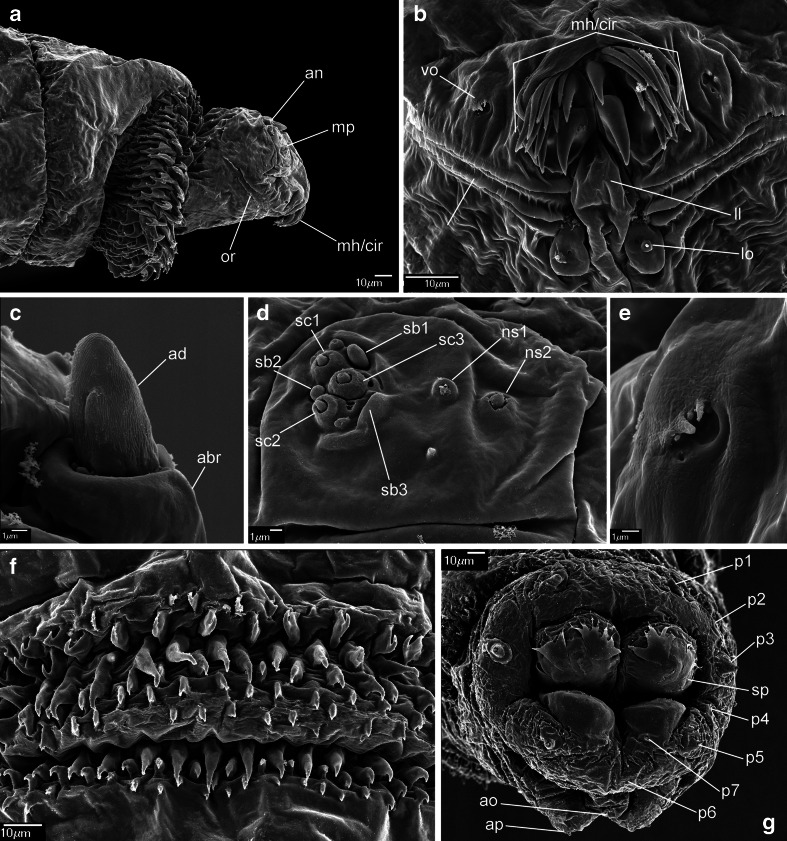
First instar of *Chrysomya bezziana*. **a** Anterior end of body, lateral view. **b** Functional mouth opening. **c** Antennal complex. **d** Maxillary palpus. **e** Ventral organ. **f** Third abdominal segment, ventral view, anterior spines. **g** Anal division, posterior view. Abbreviations: *abr* antennal basal ring, *ad* antennal dome, *an* antennal complex, *ao* anal opening, *ap* anal pad, *cir* cirri, *mh* mouthhooks, *mp* maxillary palpus, *ll* labial lobe, *lo* labial organ, *ns1* first additional sensillum coeloconicum, *ns2* second additional sensillum coeloconicum, *or* oral ridges, *p1–p7* papillae 1–7 *sb1–sb3* sensilla basiconica, *sc1–sc3* sensilla coeloconica, *sp* posterior spiracle, *vo* ventral organ

Pseudocephalon: Antennal complex with slightly elongated dome, height of basal ring less than length of antennal dome (Fig. 1c); maxillary palpus with three sensilla coeloconica and three sensilla basiconica (sc1–sc3, sb1–sb3) clustered in the central part, sb3 not distinct (Fig. 1d), one or two additional, small sensilla are situated close to sb1, two additional sensilla coeloconica (ns1, ns2) of typical appearance are arranged laterodorsally on the surface of the maxillary palpus, central cluster of sensilla surrounded by several crescent-shaped protuberances; labial lobe narrow and elongated, almost parallel-sided (Fig. 1b); ventral organ small, situated lateral to the functional mouth opening and level with the adjacent integument (Fig. 1b, e); oral ridges terminate medio-laterally on pseudocephalon (Fig. 1a). Cephaloskeleton: mouthhooks large, strongly sclerotised and equally broad for the entire length (Fig. 6a), apical part with numerous (10–13), long, strongly sclerotised, pointed teeth arranged in a large cluster and with tips orientated ventrally, three to fourth teeth of each mouthhook larger than others (Figs. 1a, b and 6a, b); labrum long, with well differentiated narrower apical part, broad basal part of labrum at most 1.5× as long as narrow apical part (Fig. 6a); intermediate sclerite H-shaped in ventral view (Fig. 6b); parastomal bars broad and straight in lateral view (Fig. 6a, b); vertical plate very wide, about three times wider than greatest width of dorsal cornua; dorsal cornua slightly longer than ventral cornua, ventral cornua at widest point 1.5× as wide as dorsal cornua (Fig. 6a); dorsal bridge present (Fig. 6b). Thoracic segments: anterior spinose band on t1 broad, with spines arranged in 7–8 rows dorsally and 12–14 rows ventrally (Figs. 1a and 6a), spines large and slightly flattened, with broad triangular base and slender, curved, hook-like tip, size of spines decreasing gradually towards the posterior end of the body (Fig. 1a); anterior spinose bands of t2–t3 with homogeneous, strongly sclerotised, slightly flattened spines, tip of spines curved. Abdominal segments: anterior spinose bands complete on a1–a5, narrowly interrupted dorsally on a6, on a7 the band is incomplete being interrupted dorsally and laterally (Fig. 7a), spines on ventro-lateral surfaces of each segment larger than other spines, each anterior spinose band ventrally with a transverse lenticular gap without spines (Fig. 1f); posterior spinose band absent on a1, on a2–a4 band present as a single row of ventral spines with few additional spines ventro-laterally, on a5 spines present as a single ventral row with an additional small group of spines laterally, a6 with posterior band interrupted dorsally and laterally, band on a7 complete with a single row of spines laterally and with 2–3 rows ventrally and dorsally (Fig. 7a). Anal division: Anal pads rounded, slightly protruding (Fig. 1g), anal tuft with several spines dorsally, readily apparent in light microscope; hair-like spines around spiracular field entirely absent (Fig. 1g), several conical spines present along ventral edge of spiracular field; posterior spiracles each with four peristigmatic tufts of similar size, tufts broad with distal margin shallowly serrated to form four to seven irregular branches; anterior spinose band developed only ventrally and ventrolaterally (Fig. 1g); p1, p3 and p5 developed as cones with a long sensillum on the extremity resembling a large sensillum coeloconica, p7 with sensillum on small protuberance, p2, p4 and p6 developed as sensilla situated level with adjacent integument (Fig. 1g).

#### *Cochliomyia hominivorax* (Coquerel, 1858) (Figs. 2a–h; 3a–d; 6c, d; and 7b

**Fig. 2:**
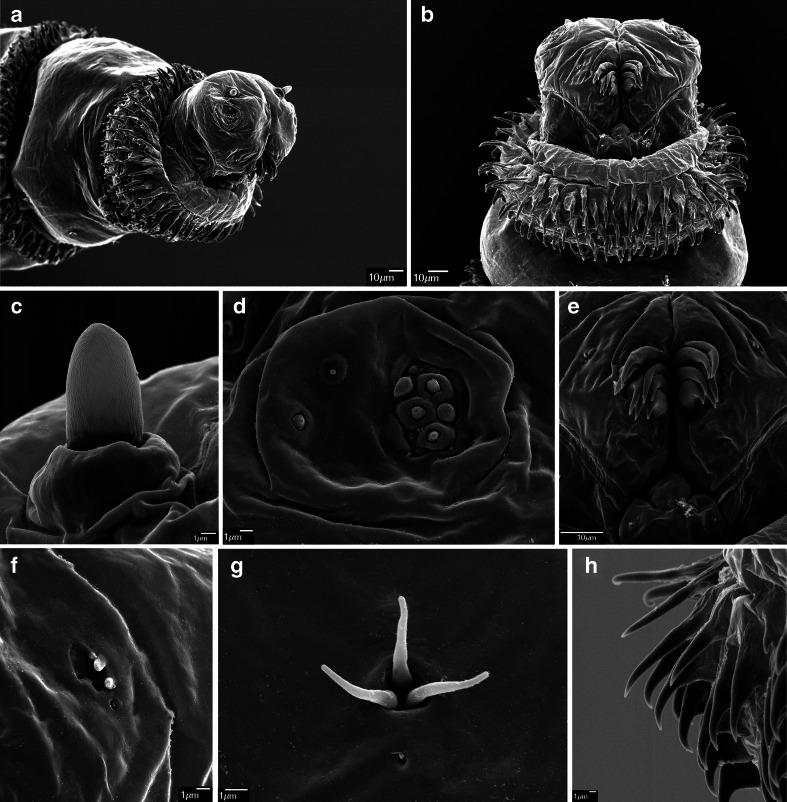
First instar of *Cochliomyia hominivorax*. **a** Anterior end of body, antero-lateral view. **b** Anterior end of body, ventral view. **c** Antennal complex. **d** Maxillary palpus. **e** Functional mouth opening. **f** Ventral organ. **g** Keilin’s organ. **h** First thoracic segment, spines

**Fig. 3:**
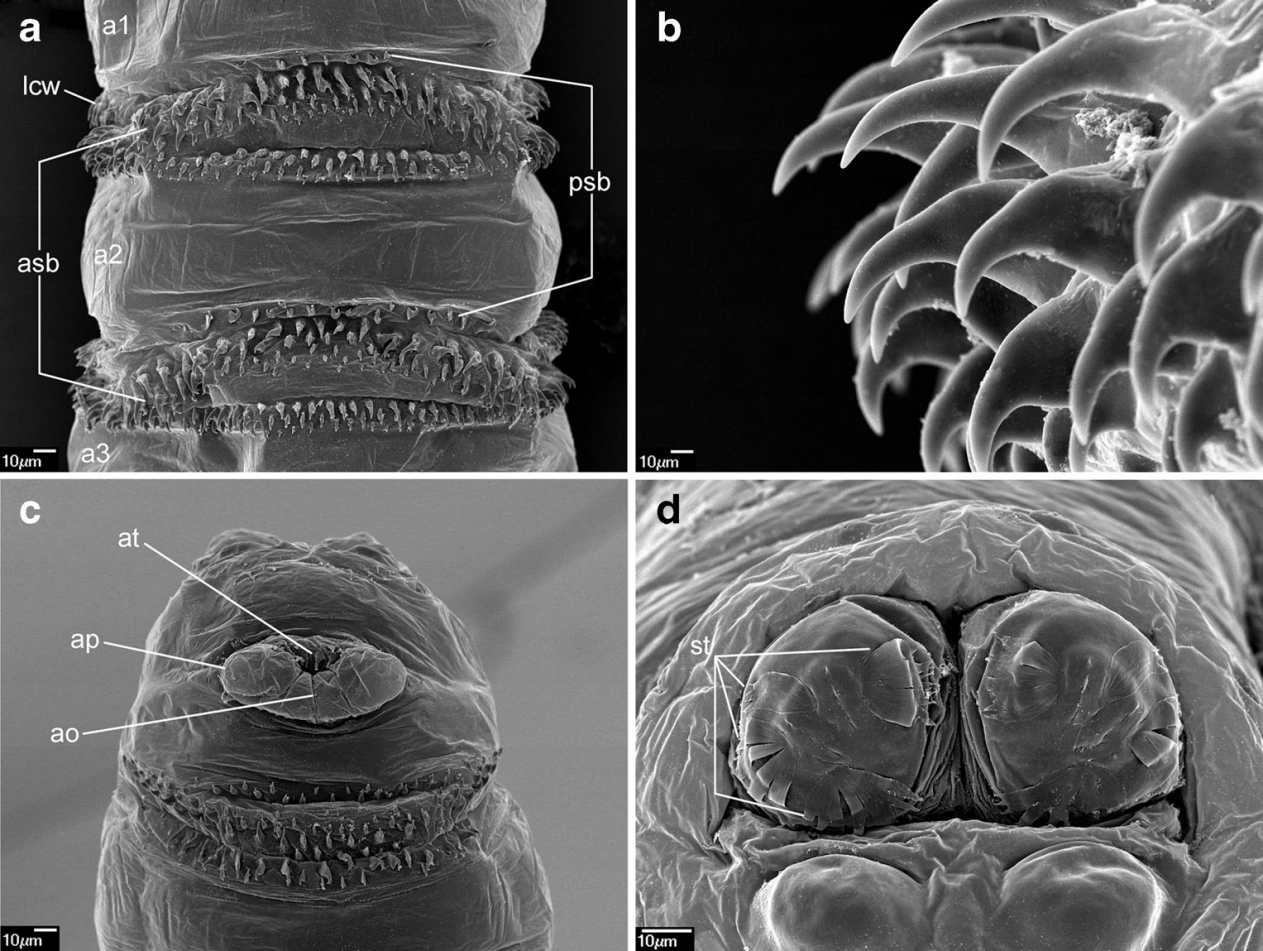
First instar of *Cochliomyia hominivorax*. **a** Abdominal segments 1–3, ventral view. **b** Abdominal segment 2, spines. **c** Anal division, ventral view. **d** Anal division, posterior spiracles. Abbreviations: *a1–a3* abdominal segments 1–3, *ao* anal opening, *ap* anal pad, *asb* anterior spinose band, *at* anal tuft, *lcw* lateral creeping welt, *psb* posterior spinose band, *st* peristigmatic tufts

Pseudocephalon: Antennal complex with slightly elongated dome, height of basal ring less than length of antennal dome (Fig. 1c); maxillary palpus with three sensilla coeloconica and three sensilla basiconica (sb1–sb3, sc1–sc3) clustered in the central part, sb3 not distinct (Fig. 2d), one or two additional small sensilla are situated close to sb1, two additional typical sensilla coeloconica (ns1–ns2) arranged laterodorsally on the surface of the maxillary palpus; labial lobe triangular (Fig. 2e); ventral organ small, situated lateral to the functional mouth opening and level with the adjacent integument (Fig. 2e, f); oral ridges terminate medio-laterally on pseudocephalon (Fig. 2a, b). Cephaloskeleton: mouthhooks strongly sclerotised in anterior and mid parts, mid part of mouthhooks bar-like, slightly curved and equally broad for the entire length, apical part with six to eight long, strongly sclerotised, pointed teeth arranged in a large cluster and with tips orientated ventrally, teeth with only slightly differentiated size, basal part of mouthhook slightly sclerotised with visible lateral arm (Figs. 2b, e and 6c, d); labrum long, with well differentiated narrower apical part, broad basal part of labrum 3× longer than short, narrow apical part (Fig. 6c); intermediate sclerite H-shaped in ventral view (Fig. 6d); parastomal bars broad and straight in lateral view; vertical plate very wide, about 3× as wide as widest part of dorsal cornua; dorsal cornua slightly longer than ventral cornua, ventral cornua with similar width to the dorsal cornua; dorsal bridge absent (Fig. 6d). Thoracic segments: anterior spinose band on t1 broad, with spines arranged in 6–7 rows dorsally and 10–12 rows ventrally, spines large and elongated, size of spines decreasing gradually towards the posterior end of body, anterior-most spines of each segment very long and almost straight with only apical part curved (Figs. 2a, b and 6c); anterior spinose bands of t2 and t3 with homogeneous, strongly sclerotised, elongated spines. Abdominal segments: anterior spinose bands complete on a1–a5, interrupted dorsally by narrow break on a6, on a7 band incomplete being interrupted dorsally and laterally (Fig. 7b), spines on ventro-lateral surfaces of each segment larger than other spines, each anterior spinose band ventrally with a transverse lenticular gap without spines (Fig. 3a); all posterior spinose bands incomplete, spines present only as single row of spines on ventral surface with few additional spines on ventro-lateral surfaces (Fig. 7b); lateral creeping welts with strong spines directed posteriorly only the most posterior lateral creeping welt without spines. Anal division: Anal pads rounded, small and slightly protruding (Fig. 3c), anal tuft with few spines dorsally, readily apparent in light microscope; circle of hair-like spines around spiracular field entirely absent (Fig. 3d), several small conical spines present along ventral edge of spiracular field; posterior spiracles each with four peristigmatic tufts of differentiated size (Fig. 3d), dorso-lateral tuft narrower than others, other tufts broad and serrated to form three to six broad branches; anterior spinose band developed only ventrally and ventrolaterally (Fig. 7b); p1, p3 and p5 developed as small cones with a short sensillum on the extremity resembling a large sensillum coeloconica, p7 with sensillum on small protuberance, p2, p4 and p6 developed as sensilla situated level with adjacent integument.

#### *Wohlfahrtia magnifica* (Schiner, 1862) (Figs. 4a–h; 5a–e; 6e, f; and 7c)

**Fig. 4:**
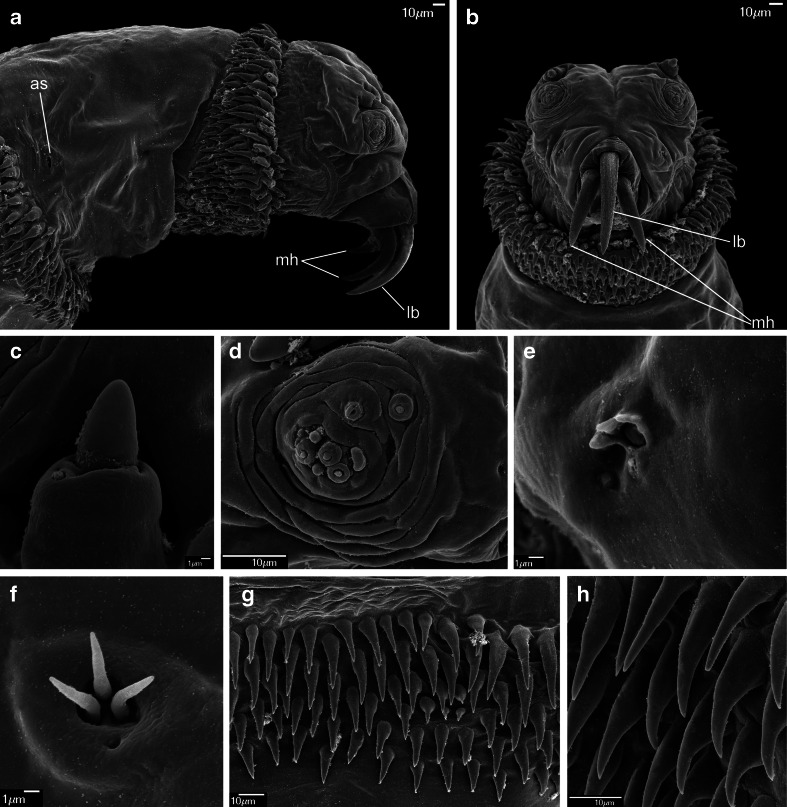
First instar of *Wohlfahrtia magnifica*. **a** Anterior end of body, lateral view. **b** Anterior end of body, ventral view. **c** Antennal complex. **d** Maxillary palpus. **e** Ventral organ. **f** Keilin’s organ. **g** Third thoracic segment, spines. **h** Second abdominal segment, spines. Abbreviations: *as* anterior spiracle, *lb* labrum, *mh* mouthhooks

**Fig. 5:**
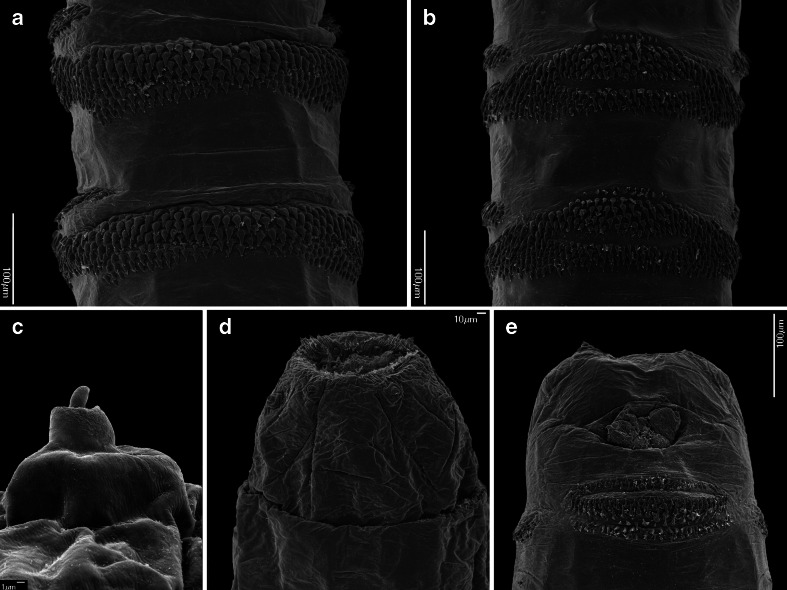
First instar of *Wohlfahrtia magnifica*. **a** Third abdominal segment, dorsal view. **b** Third abdominal segment, ventral view. **c** Anal division, papilla p5. **d** Anal division, posterior end, dorsal view. **e** Anal division, posterior end, ventral view

**Fig. 6:**
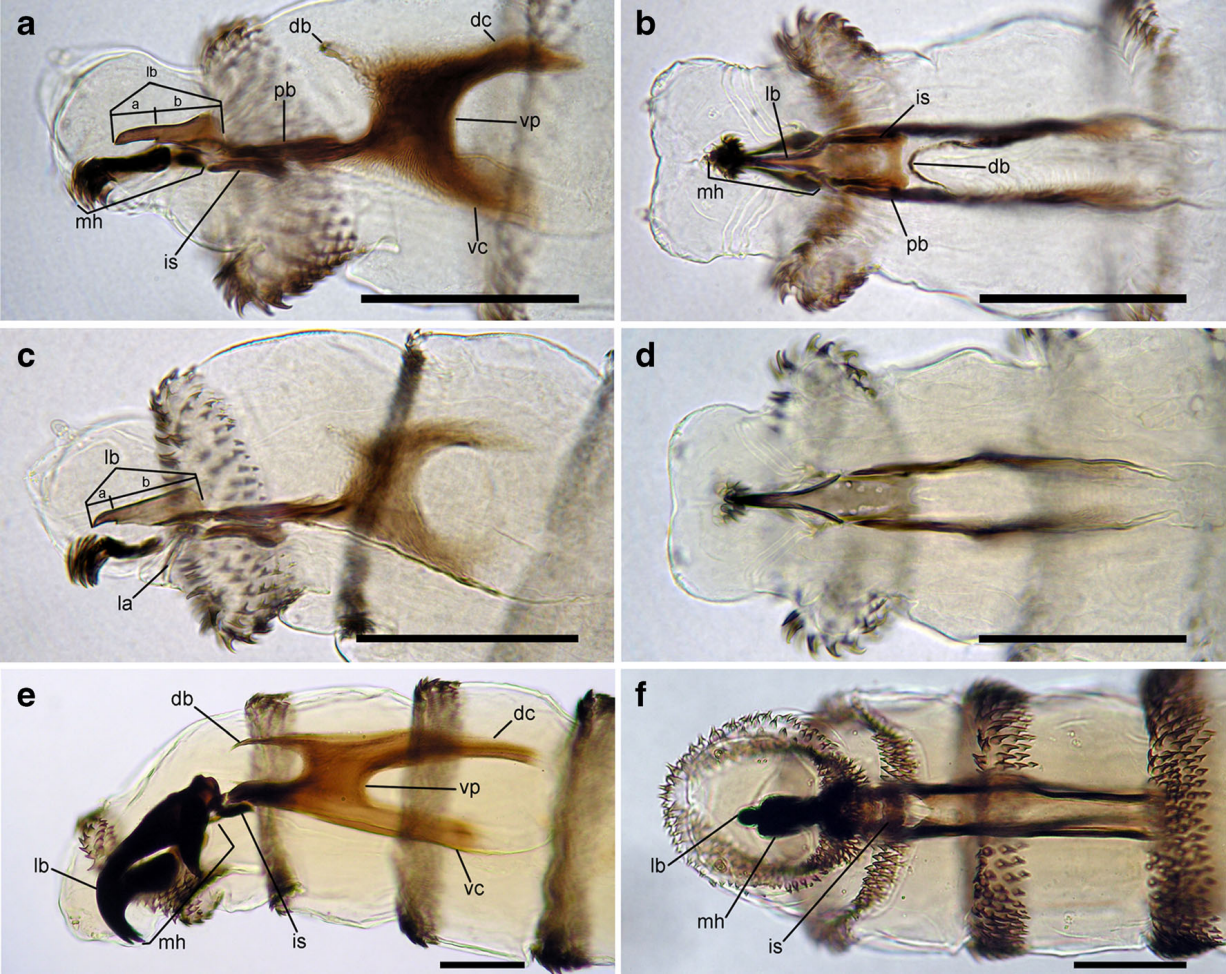
Cephaloskeleton of OTMA. **a** *Chrysomya bezziana*, lateral view. **b** *Chrysomya bezziana*, ventral view. **c** *Cochliomyia hominivorax*, lateral view. **d** *Cochliomyia hominivorax*, ventral view. **e** *Wohlfahrtia magnifica*, lateral view. **f** *Wohlfahrtia magnifica*, ventral view. Scale bar = 0.1 mm. Abbreviations: *a* length of apical part of labrum, *b* length of basal part of labrum, *db* dorsal bridge, *dc* dorsal cornua, *is* intermediate sclerite, *la* lateral arm, *lb* labrum, *mh* mouthhook, *pb* parastomal bar, *vc* ventral cornua, *vp* vertical plate

**Fig. 7:**
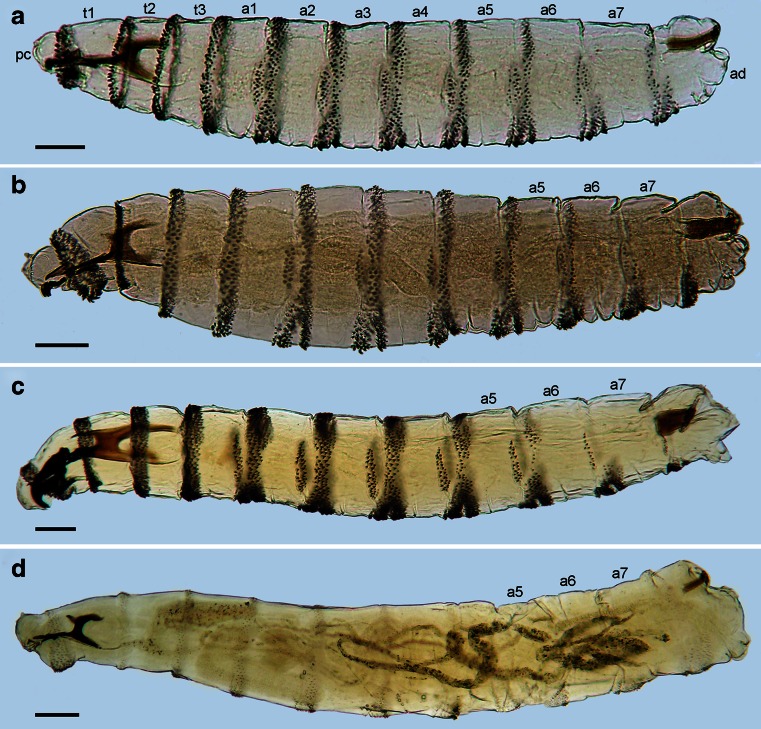
Habitus. **a** *Chrysomya bezziana*. **b** *Cochliomyia hominivorax*. **c** *Wohlfahrtia magnifica*. **d** *Lucilia cuprina*. Abbreviations: *a1–a7* abdominal segments 1–7, *ad* anal division, *pc* pseudocephalon, *t1–t3* thoracic segments 1–3. Scale bar = 0.1 mm

Pseudocephalon: Antennal complex with slightly conical dome, height of basal ring greater than length of antennal dome (Fig. 4c); maxillary palpus encircled by several cuticular folds, central cluster of sensilla with three sensilla coeloconica and three sensilla basiconica (sb1–sb3, sc1–sc3) (Fig. 4d), few other small sensilla are situated close to sb1, two additional sensilla coeloconica (ns1–ns2) of typical appearance arranged laterodorsally on the surface of the maxillary palpus (Fig. 4d); ventral organ small, situated lateral to the functional mouth opening and level with the adjacent integument (Fig. 4b, e); oral ridges terminate medio-laterally on pseudocephalon (Fig. 4a). Cephaloskeleton: mouthhooks large and strongly sclerotised, anterior part of each mouthhook strongly curved downward and with single pointed tip, basal part with well visible lateral arm, tips of teeth orientated ventrally (Figs. 4a, b and 6e); labrum very large and long with massive basal part, anterior part of labrum strongly curved downward appearing to represent a third, middle mouthhook (Figs. 4a, b and 6e); intermediate sclerite short, partly hidden behind parastomal bar in lateral view but clearly shifted toward anterior end of body under base of labrum (Fig. 6e, f); parastomal bars short and broad (Fig. 6e, f); vertical plate wide, about three times wider than width of ventral cornua; dorsal cornua longer than ventral cornua, but both cornua of similar width (Fig. 6e); dorsal bridge present. Thoracic segments: anterior spinose band on t1 broad, with spines arranged in 5–6 rows dorsally and 9–11 rows ventrally, spines very large more conical than in *Chrysomya bezziana*, elongated and slightly curved, size of spines decreasing gradually towards the posterior end of body (Fig. 4a, b); anterior spinose bands of t2 and t3 with homogeneous, strongly sclerotised, elongated spines. Abdominal segments: anterior spinose bands complete on a1–a5, on a6 band narrowly interrupted dorsally, on a7 the band incomplete, restricted to ventral surface and few spines on dorso-lateral surfaces, each anterior spinose band ventrally with a transverse lenticular gap without spines (Figs. 5b and 7c); posterior spinose band on a1–a6 band present as a single row of ventral spines with few additional spines ventro-laterally, band on a7 complete with a single row of spines on lateral surfaces and with 2–3 rows ventrally and dorsally; lateral creeping welts with strong spines directed posteriorly, only the most posterior lateral creeping welt without spines. Anal division: Anal pads rounded, small and slightly protruding (Fig. 5e), anal tuft with several spines dorsally, readily apparent in light microscope; hair-like spines around spiracular cavity present but sparse (Fig. 5d); posterior spiracles hidden in spiracular cavity (Fig. 5d); p1, p3 and p5 developed as large cones with a long sensillum on the extremity resembling a large sensillum coeloconicum (Fig. 5c, d), p7 with sensillum on small protuberance, p2, p4 and p6 developed as sensilla situated level with adjacent integument.

## Key to the first instar larva of obligatory traumatic wound myiasis agents of domestic animals and humans

The following keys will serve to separate the first instar of the three known OTMAs from other calyptrates and reliably identify them to species:

1. Posterior spiracular cavity absent, spiracles exposed on a flat or slightly concave spiracular field (Figs. 1g and 3d)…2
  Deep spiracular cavity present, posterior spiracles hidden (Fig. 5d)…4
2. Teeth of mouthhooks and cirri strong, large and curved (Figs. 1b, 2e and 6a, c); oral ridges terminate medio-laterally on pseudocephalon (Figs. 1a and 2a), spines of spinose bands relatively large and strongly sclerotised (Fig. 7a, b), hair-like spines around spiracular field absent (Figs. 1g and 3d), peristigmatic tufts with broad and short branches, cleft at most to centre of tuft (Figs. 1g and 3d)…3
  Teeth of mouthhooks and cirri distally slender and mostly straight (see Szpila et al. 2013, Fig. 3e), oral ridges terminate dorso-laterally on pseudocephalon (see Szpila et al. 2013, Fig. 3a, b), spines of spinose bands relatively small and weakly sclerotised (Fig. 7d), hair-like spines around spiracular field present, branches of peristigmatic tufts long and slender, cleft almost to base of tuft (see Szpila et al. 2013, Fig. 4d, e, h)…(other Calliphoridae)
3. Mouthhooks with apical teeth clearly differentiated in size with three to four teeth of each mouthhook larger than remaining teeth/cirri (Fig. 1b), basal part of labrum about 1.5× longer than narrow apical part (Fig. 6a), dorsal bridge present (Fig. 6b), posterior spinose band on segment a7 complete (Fig. 7a)…*Chrysomya bezziana*
  Mouthhooks with apical teeth only slightly differentiated in size (Fig. 2e), basal part of labrum at least 3× longer than narrow apical part (Fig. 6c), dorsal bridge absent (Fig. 6d), posterior spinose band on segment a7 incomplete (Fig. 7b) … *Cochliomyia hominivorax*
4. Labrum of cephaloskeleton well developed, distinctly visible between the paired mouthhooks (Figs. 4a, b; 6e–f)…5
  Labrum of cephaloskeleton vestigial, only the paired mouthhooks well visible…Sarcophaginae
5. Anterior part of labrum and mouthhooks abruptly and almost perpendicularly curved downward (Fig. 6e), spines of spinose bands large and strongly sclerotised (Fig. 7c), posterior spinose bands on a5–a6 incomplete, interrupted dorsally (Fig. 7c)…*W. magnifica*
  Anterior part of labrum and mouthhooks gently and gradually curved downward (see Szpila and Villet 2011, Fig. 10j), spines of spinose bands small and weakly sclerotised, posterior spinose bands on a5–a6 complete…(other Paramacronychiinae)

## Discussion

### Verification of earlier descriptions

Existing data on L1 morphology of OTMA species are very uneven with regard to the quality of the illustrations and the accuracy of the descriptions. The first report of the L1 of *Chrysomya bezziana* provided by Zumpt (1965) is only a vague description of the general appearance of larvae with a note about the serrated tips of the mouthhooks. More comprehensive documentation was published by Kitching (1976), but several misinterpretations of the distribution and composition of the spinose bands are obvious. Firstly, Kitching (1976) treated the anterior spinose band of t1 as an integral part of the pseudocephalon (head segment with a posterior ruff-like structure with five to six rows of irregularly aligned spines). An indirect consequence of this interpretation is the erroneous statement that thoracic and abdominal segments possess only a posterior spinose band. True posterior spinose bands on abdominal segments are interpreted as “one row of these smaller spines along the anterior margins of spine bands”. However, the SEM micrograph of the ventral abdominal surface presented by Kitching (1976, Fig. 23) shows that the larval spinulation analysed by him was of the same type as that described in the present paper, with a broad anterior spinose band and a narrow incomplete posterior spinose band. Gan (1980) presented only a very short description of the cephaloskeleton, but it was supported by a very good line drawing. This figure of the cephaloskeleton is fully congruent with the cephaloskeleton of *Chrysomya bezziana* as presented here. Spradbery (2002), in a manual for diagnosis of screw-worm flies, provided only a superficial description of the L1 of *Chrysomya bezziana* without any details.

Documentation of the L1 morphology available for *Cochliomyia hominivorax* is generally much better, mostly thanks to Laake et al. (1936). These authors provided a detailed description of the cephaloskeleton and spinulation, line drawings of the cephaloskeleton and a habitus of the entire larva. All details of their documentation are congruent with the data presented here. Noteworthy is the contribution of Lopes (1983), with a figure of the anterior part of the cephaloskeleton of the L1 of *Cochliomyia hominivorax*. Interestingly, Lopes (1983) interpreted the apical teeth of the mouthhook (conspicuous series of spines on the margins of oral atrium) as structures separated from mouthhooks (maxilla). This kind of interpretation was common in previous descriptions of the L1 of blowflies (Szpila et al. 2008). However, the strongly sclerotised mouthhook of *Cochliomyia hominivorax* provides firm evidence (see Fig. 6c, d) that at least some of these exposed teeth are an integral part of the mouthhook. The next major contribution to larval morphology of *Cochliomyia hominivorax* was published by Leite and Guevara (1993) and was based mostly on SEM micrographs. In comparison to the present data only one important difference was found: Leite and Guevara (1993) reported the presence of three peristigmatic tufts on each posterior spiracle, but SEM micrographs in their own publication (Leite and Guevara 1993, Figs. 11 and 12) and in the present paper (Fig. 3d) all show four peristigmatic tufts. A technical mistake is apparent in Fig. 10 of Leite and Guevara (1993) where the anal division is presented upside down and the anal opening (k) is marked between the posterior spiracles instead of between the anal pads.

Valentyuk (1971) presented details of the L1 of *W. magnifica* in the context of an identification key to larvae of *Wohlfahrtia* of Crimea and the north coast of the Black Sea. The data provided are sparse but refer to characters of significant taxonomic importance: the shape of mouthhooks and labrum; and the size, sclerotisation and distribution of spines on the segments a6–a7. One difference noted between the data of Valentyuk (1971) and those presented here concerns the posterior spinose band on a7. In our material, the posterior spinose band encircles all of a7 (in the form of a single row of spines both laterally and dorsally) whereas Valentyuk (1971) reported spines to be entirely absent on the dorsal surface of a6–a7 and on the anal division. A detailed study of the L1 of *W. magnifica* provided by Schumann et al. (1976) is largely congruent with the present data regarding the shape of the cephaloskeleton and details of spinulation. One discrepancy concerns the degree of development of the anterior spinose band on the anal division. Schumann et al. (1976) described it as incomplete, similarly to Valentyuk (1971). Lehrer and Fromunda (1986) also provided valuable documentation of the L1 of *W. magnifica*. Their contribution is mostly restricted to characters of the cephaloskeleton and is fully congruent with the present data. The first extensive study of the larval morphology of *W. magnifica* was published by Ruiz-Martinez et al. (1989, 1990). Unfortunately, these publications contain a few significant misinterpretations: (1) the labrum is treated as a third mouthhook “identical to the lateral ones, morphologically and ultrastructurally”; (2) the intermediate sclerite is interpreted as a “parastomal sclerite of interinstar I–II”; (3) the dorsal bridge (as “dorsal arc”) of the cephaloskeleton is absent; and (4) the spines of the anterior spinose band on t1 are interpreted as “modified buccal spines”. An unpaired labrum situated between the paired mouthhooks is typical for the L1 of most Oestroidea (e.g. Grunin 1955; Ferrar 1987; Szpila 2010) except for larvae of Oestrinae (Grunin 1957), Sarcophaginae (Lopes 1983) and Rhinophoridae (Bedding 1973; Pape and Arnaud 2001). Presence of a large hook-like labrum seems to be characteristic for species of Paramacronychiinae (e.g. Thompson 1921; Valentyuk 1971; Lopes 1983; Verves 1988) and should not be treated (even in a functional approach) as a “third mouthhook”. The interpretation of the presence of a fully formed dorsal bridge is still rather open—it is possible that this structure is weakly sclerotised in early first instar larvae and that it may be interpreted as the lack of a fully developed dorsal bridge. The presence of “modified buccal spines” as described by Ruiz-Martinez et al. (1989) appears to be a misinterpretation. No “buccal spines” are reported or documented on the SEM micrographs of the L1 of *W. magnifica* by Ruiz-Martinez et al. (1990) in a paper published one year later, nor on the SEM images in the present paper. The description of Ruiz-Martinez et al. (1989, 1990) of the L1 spinulation is superficial and with the only taxonomically valuable information that the spines are strongly sclerotised. The mention by Ruiz-Martinez et al. (1989) of the presence of a “triangular sclerotised plate between the dorsal and ventral cornua (Fig. 1a, h)” as a character of significant taxonomic importance is of interest. This structure was not present in any larvae in our material. Ruiz-Martinez et al. (1990) also stated that the “mouthhooks are always projected […] because their dimension limit the retraction into cephalic segment”. This is an apparent misinterpretation—specimens of *W. magnifica* with entirely retracted cephaloskeletons are presented in the present publication (Fig. 6c). The large and elongated labrum may be fully retracted also in the first instar of *Sarcophila latifrons* (Fallén, 1817) as well as in some (probably most) species of Miltogramminae (Szpila 2010).

### Convergent morphology in OTMA species

Species of OTMA belong to quite distantly related taxonomic units. *Chrysomya bezziana* and *Cochliomyia hominivorax* are classified in the same subfamily Chrysomyinae, but are situated on relatively distant branches of the phylogenetic tree of Calliphoridae (McDonagh and Stevens 2011). *Wohlfahrtia magnifica* represents a separate family, Sarcophagidae. Morphological and molecular data strongly support an independent origin of obligatory parasitism for each of the OTMA species (Hall et al. 2009; McDonagh and Stevens 2011). Consequently, the distinct morphological similarities can only be explained as convergent evolution and may be suspected to be homoplasic adaptations for feeding in living vertebrate tissues. Shared character states of the first instar of OTMA species not found in closely related necrophages and facultative parasites are the following: (1) strongly sclerotised anterior structural elements of the cephaloskeleton; (2) teeth of mouthhooks and cirri apically strongly curved, hook-like; (3) spinose bands on t1–t3 and a1–a7 with strongly sclerotised, enlarged spines; (4) reduction of spinose bands on segments a6, a7 and anal division. The L1 of *Chrysomya bezziana* and *Cochliomyia hominivorax*, being more closely related to each other than to *W. magnifica*, possess further convergent specific character states in comparison to necrophagous and facultative parasitic species, i.e. (1) shortening of oral ridges, which terminate medio-laterally on the pseudocephalon; (2) absence of hair-like spines around the spiracular field on the anal division; and (3) peristigmatic tufts with broad and sparsely dichotomous branches.
